# Global coordination level in single-cell transcriptomic data

**DOI:** 10.1038/s41598-022-11507-y

**Published:** 2022-05-09

**Authors:** Guy Amit, Dana Vaknin Ben Porath, Orr Levy, Omer Hamdi, Amir Bashan

**Affiliations:** 1 Physics Department, Bar-Ilan University, Ramat-Gan, Israel; 2 Department of Immunobiology, Howard Hughes Medical Institute, Yale University School of Medicine, New Haven, CT USA

**Keywords:** Gene regulatory networks, Transcriptomics, RNA sequencing

## Abstract

Genes are linked by underlying regulatory mechanisms and by jointly implementing biological functions, working in coordination to apply different tasks in the cells. Assessing the coordination level between genes from single-cell transcriptomic data, without a priori knowledge of the map of gene regulatory interactions, is a challenge. A ‘top-down’ approach has recently been developed to analyze single-cell transcriptomic data by evaluating the global coordination level between genes (called GCL). Here, we systematically analyze the performance of the GCL in typical scenarios of single-cell RNA sequencing (scRNA-seq) data. We show that an individual anomalous cell can have a disproportionate effect on the GCL calculated over a cohort of cells. In addition, we demonstrate how the GCL is affected by the presence of clusters, which are very common in scRNA-seq data. Finally, we analyze the effect of the sampling size of the Jackknife procedure on the GCL statistics. The manuscript is accompanied by a description of a custom-built Python package for calculating the GCL. These results provide practical guidelines for properly pre-processing and applying the GCL measure in transcriptional data.

## Introduction

Most of the functions of living cells are carried out by interacting genes, which are dictated by delicate gene regulatory networks^1,2^. Characterizing these networks and their relation to different phenotypes is of great scientific interest^3,4^. Recent progress in experimental and bioinformatical techniques provides large datasets of single-cell transcriptomic profiles and have the potential to advance the study of gene regulatory networks associated with specific tissues, cell types and conditions^5–9^. However, complete reconstruction of the gene regulatory network is still a major challenge, not only because of the huge number of functional genes^10–12^, but also because of the inherent stochasticity of such systems^13^. Even so, certain important and general features of this network can sometimes be extracted without fully inferring it. For example, the network connectivity, i.e., the density of actual among all possible gene–gene interactions, may have important implications for general processes in the cells, as it can be related to the percolation properties of the network which affects signal transmission^2^. Furthermore, in several processes, such as aging or cancer, cellular gene regulatory networks are subject to alterations that are stochastic in nature, so the affected elements are different across individual cells of the same tissue^14^. In such cases, the tissue-level effect may be captured better by general measures than by detailed network inference.

To tackle this difficulty we have recently introduced the Global Coordination Level (GCL) measure in the context of aging research^15^. Using this new method, we were able to show that the inherent stochastic processes in aging cells are associated with a decrease in the coordination between genes, a pattern that is observed consistently across different cell types and organisms. The GCL measures the average multivariate dependency between the expression levels of random subsets of genes in single-cell RNA-seq data. A key advantage of the GCL is that it is a *top-down* measure, i.e., it does not require the complete reconstruction of the gene regulatory network. This is in contrast to network analysis of the gene regulatory relations extracted in a *bottom-up* approach, e.g., using co-expression analysis^1^. By avoiding the network reconstruction, the GCL also has the advantage that it does not assume pair-wise interactions or a specific form of relation (e.g., linear relations), and it does not assume the same type of interaction for all the pairs of interacting genes. The GCL was also found useful in distinguishing between cell-to-cell variability induced by biological process and variability induced by technical noise in simulations of cellular networks^16^.

In this manuscript, we systematically investigate several key aspects of the GCL and, based on it, provide precise instructions and recommendations on how to use this method on scRNA-seq data, as well as the pre-processing filtering steps required for it and recommended parameters selections. As mentioned, the GCL works by calculating the general multivariate dependencies between the genes for a cohort of cells. It does so by a repeated procedure of randomly selecting gene subsets and calculating the distance correlation between them^17^. Averaging over many such subsets of genes yields a single numerical value (the GCL) which evaluates the total dependencies between the genes. Before doing so however, there are several pre- and post-processing steps which must be taken into account. There are three major steps: (1) Clustering cells into homogeneous groups. (2) Filtering cells that differ significantly from the other cells (‘outliers’), or cells which are too similar to each other (‘inliers’). (3) Performing jackknife analysis to ensure the stability of the results. These steps are required because, as we show, the GCL (like other correlation measures) is sensitive to unusual cells and heterogeneous cohorts, while scRNA-seq data is typically sparse, noisy and characterized by outliers and clusters. Based on a systematic analysis of the effect of the above steps, the recommended guidelines presented here are important to ensure stable and reliable results of the GCL measure.

## Material and methods

### Global coordination level (GCL)

As mentioned in the introduction, the GCL is a ‘top-down’ computational method to evaluate the system-wide multivariate dependency between the genes, by calculating the distance correlation of random gene sets. A set of *M* measured cells with *N* genes is represented by the matrix $X\in {\mathbb {R}}^{N\times M}$, where each vector column ${\mathbf{}}{x}^{(\nu )}(\nu =1\dots M)$ is the measurement of an individual cell $\nu$, and each element within the vector $x_i^{\nu } (i=1\dots N)$ is the measured expression value of gene *i*. The GCL is then performed as follows. The matrix *X* is divided into two random complementary parts *G* and $\overline{G}$. The two parts are represented by the matrices $X_G\in \mathbb {R}^{N/2 \times M}$ and $X_{\overline{G}}\in \mathbb {R}^{N/2 \times M}$ such that $X_G$ represents one randomly selected subset of the genes of size *N*/2, while $X_{\overline{G}}$ represents the rest of the genes ($G\cap \overline{G}=\varnothing$ and $|G \cup \overline{G}|=N$). Then, we calculate the bias-corrected distance correlation (bcdCorr)^17^ between groups *G* and $\overline{G}$. The bcdCorr, a refined version of the distance correlation (dCorr) measure^18^, evaluates the level of dependence between two high-dimensional variables by testing how the distance between the samples, with respect to one (high-dimensional) variable, is changed compared to the distance between the same samples with respect to the other (high-dimensional) variable. Accordingly, when applied to gene-expression data, the bcdCorr is a measure of the dependency level between the gene-sets *G* and $\overline{G}$. A short description of how to calculate the bcdCorr between two variables is presented below. The process of calculating the bcdCorr is repeated *m* times, for different random divisions of the genes. Finally, the GCL is defined as the average across these divisions, i.e.,

1 $$\begin{aligned} \mathrm {GCL}(X) = \frac{1}{m}\sum _{k = 1}^m \mathrm {bcdCorr}(X_G^k, X^k_{\overline{G}}), \end{aligned}$$

where all *m* random divisions of the data $(X_G^k, X^k_{\overline{G}})$ are independently chosen. For a large homogeneous cohort, the empirical GCL is bounded between zero and one: zero corresponds to the case of independent gene expression, whereas a significantly non-zero GCL reflects coordinated transcriptional expression, which could be interpreted as a result of underlying molecular dynamics, such as gene-to-gene regulatory interactions.

### Calculating the bcdCorr

Here we briefly show the full procedure of calculating the empirical bcdCorr between two variables as defined by Székely and Rizzo^17^. Consider *M* observations of two high-dimensional variables $X_i\in \mathbb {R}^p$ and $Y_i\in \mathbb {R}^q$, $i=1,\dots ,M$, where $X_i=(X_{i, 1}, \dots , X_{i,p})$ and $Y_i=(Y_{i, 1}, \dots , Y_{i, q})$. Note that *q* does not necessarily have to be equal to *p* (i.e., the two variables may have unequal dimensions). The *M* observations are represented by the $p\times M$ matrix *X* and $q\times M$ matrix *Y*. The empirical $\mathrm {bcdCorr}(X, Y)$ is defined as

2 $$\begin{aligned} \mathrm {bcdCorr}(X, Y) = \frac{\mathrm {dCov}(X, Y)}{\sqrt{\mathrm {dCov}(X, X)\times \mathrm {dCov}(Y, Y))}}, \end{aligned}$$

where

3 $$\begin{aligned} \mathrm {dCov}(X, Y) = \frac{1}{M(M-3)}\left[ \sum _{i, j=1}^M A^*_{i, j}B^*_{i, j} - \frac{M}{M-2}\sum _{i=1}^M A^*_{i, i}B^*_{i, i} \right] , \end{aligned}$$

and $A^*_{i, J}$ and $B^*_{i, j}$ are matrices defined as

4 $$\begin{aligned} A^*_{i, j} = {\left\{ \begin{array}{ll} \frac{M}{M-1}\left( A_{i, j} - \frac{a_{ij}}{M} \right) , &{} i\ne j\\ \frac{M}{M-1}(\bar{a}_i - \bar{a}), &{} i=j \end{array}\right. }, \quad B^*_{i, j} = {\left\{ \begin{array}{ll} \frac{M}{M-1}\left( B_{i, j} - \frac{b_{ij}}{M} \right) , &{} i\ne j \\ \frac{M}{M-1}(\bar{b}_i - \bar{b}), &{} i=j \end{array}\right. }. \end{aligned}$$

$A_{i, j}$ and $B_{i, j}$ are matrices defined as

5 $$\begin{aligned} \begin{aligned} A_{i, j}&= a_{ij} - \bar{a}_i - \bar{a}_j + \bar{a}, \\ B_{i, j}&= b_{ij} - \bar{b}_i - \bar{b}_j + \bar{b}, \end{aligned} \end{aligned}$$

where

6 $$\begin{aligned} a_{ij}= & {} \left\Vert X_i - X_j\right\Vert ,\quad i,j = 1,\dots ,M, \end{aligned}$$

7 $$\begin{aligned} a_{i\cdot }= & {} \sum _{k=1}^M a_{ik}, \quad a_{\cdot j} = \sum _{k=1}^m a_{kj}, \quad \bar{a}_i = \bar{a}_{i\cdot } = \frac{1}{n}a_{i\cdot }, \end{aligned}$$

8 $$\begin{aligned} a_{\cdot \cdot }= & {} \sum _{i, j=1}^M a_{ij}, \quad \bar{a}=\frac{1}{n^2}\sum _{i, j=1}^M a_{ij}, \end{aligned}$$

$\left\Vert X\right\Vert = \langle X, X\rangle ^{1/2}$ is the Euclidean norm and $b_{ij}$, $\bar{b}_i$, $\bar{b}_j$ and $\bar{b}$ are defined similarly for *Y*. It can be shown that this estimator for the distance correlation is unbiased with repsect to the dimensionality of $X_i$ and $Y_i$, namely, *q* and *p*.

### Clusters detection

Methods of identifying and removing clusters from the data are numerous and well studied. In this manuscript, we advise to apply detection and removal of clusters in the following manner. We apply the *k*-means algorithm, with varying the total number of clusters, $1<K<10$. The distance between the samples is measured using the Spearman dissimilarity.

We follow that by a Silhouette coefficient analysis that determines the optimal number of clusters. For each of the $C_k$ clusters ($k=1\dots K$), we calculate the mean distance, $a_i$, between each cell ${\mathbf{}}{x}^i$ and all the other cells which belong to the same cluster, ${\mathbf{}}{x}^j$ ($j\in C_k, j\ne i$), namely,

9 $$\begin{aligned} a_i = \frac{1}{\left|C_k\right|-1}\sum _{j\in C_k, i\ne j}\mathrm {Spearman}({\mathbf{}}{x}^i, {\mathbf{}}{x}^j), \end{aligned}$$

where $\left|C_k\right|$ is the number of samples in cluster *k*, and $\mathrm {Spearman}({\mathbf{}}{x}^i, {\mathbf{}}{x}^j)$, is the distance between cell *i* and *j* based on the Spearman dissimilarity. Additionally, we calculate the smallest mean distance between cell *i* to all other cells in the other clusters, of which *i* is not a member, $b_i$,

10 $$\begin{aligned} b_i = \min _{k'\ne k} \frac{1}{\left|C_{k'}\right|}\sum _{j\in C_{k'}}\mathrm {Spearman}({\mathbf{}}{x}^i, {\mathbf{}}{x}^j). \end{aligned}$$

The silhouette score of each cell, $s_i^K$ ($i=1\dots M$) is then defined as

11 $$\begin{aligned} s_i^K = {\left\{ \begin{array}{ll} 1-a_i/b_i, &{} \text {if }\; a_i<b_i \\ 0, &{} \text {if }\; a_i = b_i\\ b_i/a_i-1, &{} \text {if }\; a_i>b_i, \end{array}\right. } \end{aligned}$$

where the superscript *K* indicates that the value is calculated for this particular choice of total number of clusters. nThe value of the silhouette score is bounded $-1<s_i^K<1$. Values which are close to 1 means that the data is clustered correctly with respect to cell *i*. We average the silhouette value across all the cells to get a unified value

12 $$\begin{aligned} S^K = \frac{1}{M}\sum _{i=1}^M s_i^K. \end{aligned}$$

Finally, the optimal value of *K* is chosen by maximizing the value of $S^K$ with 10 realizations of the *k*-means algorithm. In general, as each clustering detection method has its own advantages and disadvantages, we recommend supplementing the detection step with a visual inspection using dimensional reduction analysis, such as *t*-SNE.

### Effect of an individual cell on the GCL

**Figure 1:**
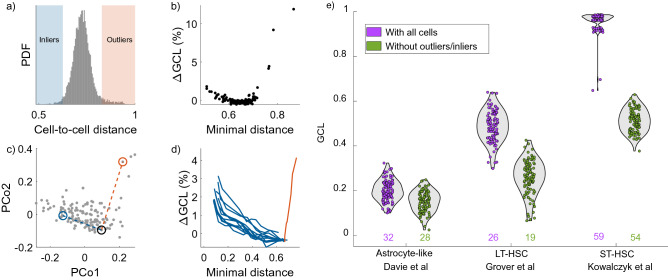
The effects of outliers and very similar cells (‘inliers’) on the GCL. The GCL value of ST-HSCs from young mice^19^ is analyzed with respect to the effects of individual outlier and inlier cells. (**a**) Proposed procedure of characterizing inliers and outliers. The histogram represents the distribution of cell-to-cell ‘distance’, measured as the Spearman dissimilarity, among all the cells. The blue and red shaded areas represent distance values smaller or larger than two standard deviations from the mean, respectively. (**b**) The effect on the GCL of each cell is measured with respect to its minimal distance to any other cell. The effect on the GCL is measured by the difference between the GCL values calculated with and without the cell (Eq. 13). Cells with unusually high or low minimal distance (outliers or inliers) have the most effect on the change in the GCL. (**c**) PCA plot of the cells. The dashed lines represent the trajectories simulated cells undergo as they transform from an average cell to an inlier cell (blue line), or to an outlier (red line). (**d**) Examples of introducing a simulated inlier or outlier cell into the real data. Each blue line represents the effect of a simulated cell which is a linear combination between an inlier and an average cell (10 simulations in total). As the distance between it and another cell decreases, its effect on the GCL increases. The red line represents a similar procedure for the outlier case. (**e**) GCL values of three different datasets from Refs.^19,21,22^ (see “Material and methods” section). Each dot represents an individual jackknife procedure, and the grey violin plots represent the distribution of values. For the ST-HSC case, the cells are from old mice. The number of cells before and after filtering is written below, with the corresponding colors. The presence of outliers/inliers tends to increase the GCL value, with a bimodel distribution apparent in the ST-HSC case where the effects of the outliers are especially pronounced. The GCL value before and after removal of the outliers/inliers was changed by the following values $23\%$, $46\%$, $47\%$ (corresponding to the ordering of the figure). We removed obvious clusters from the Astrocyte-like and LT-HSC clusters (see The effect of clusters section). The number of jackknife realizations in each case is 100, with each realization containing $70\%$ of the cells in the cohort. In all cases, the number of random divisions, *m*, is equal to 50, and the top $N=2000$ expressed genes are analyzed.

In “Removal of abnormal cells” section of this manuscript, we measure the effect of a single outlier or inlier on the GCL value. The relative effect $\Delta GCL_i$ of cell *i* on the cohort of cells is defined as

13 $$\begin{aligned} \Delta \mathrm {GCL}_i = \frac{\mathrm {GCL\ with\ cell\ } i- \mathrm {GCL\ without\ cell\ } i}{\mathrm {GCL\ with\ cell\ } i}, \end{aligned}$$

where ‘$\mathrm {GCL\ with\ cell\ } i$’ is the GCL of the entire cohort and ‘$\mathrm {GCL\ without\ cell\ } i$’ is the GCL of the cohort but with cell *i* excluded. In general, outliers and inliers tend to increase the GCL value of the cohort (see e.g. Figure 1), making the $\Delta GCL_i$ positive.

### Transcription data sets used in this work

In the demonstration presented here we have used single-cell RNA-seq datasets from the following sources (Table 1).

**Table 1:** List of the data-sets used in this work. The data-sets are freely available online and the experimental and bioinformatics processes are detailed in the original publications.

Organism	Tissue	Cell type	Study	Ages	Sex
Mouse, C57BL/6	HSCs	MPP-HSCs	Kowalczyk et al.^19^	2/24 months	Females
Mouse, C57BL/6	HSCs	ST-HSCs	Kowalczyk et al.^19^	2 months	Females
Mouse, C57BL/6J	HSCs	MPP-HSCs	Mann et al.^20^	2–3/20–24 months	Males and females
Mouse, C57BL/6J	HSCs	LT-HSCs	Grover et al.^21^	2–3 months	Males and females
*Drosophila*	Brain	Astrocyte-like cells	Davie et al.^22^	1 day	Males and females

### Numerical model for synthetic gene expression data

In the Results we investigate the effect of the presence of clusters in the data on the GCL value with synthetic gene expression data obtained from simulated numerical models of gene regulatory dynamics. The expression profile ${\mathbf{}}{x}^{(\nu )}$ of cell $\nu$ is modelled as the steady state of a set of coupled ordinary differential equations (ODEs), representing the gene regulatory dynamics^23–25^. Specifically, we use the following set of ODEs,

14 $$\begin{aligned} \dot{\varvec{x}}^{(\nu )}_i=-\varvec{x}^{(\nu )}_i+\sum _{j}w_{i,j}\frac{\varvec{x}_j^{(\nu )}}{1+\varvec{x}_j^{(\nu )}}\quad i,j\in 1\dots N, \end{aligned}$$

where we set the number of genes, $N=200$. The first term expresses a self degradation of gene *i*. The second term is responsible for the growth of $\varvec{x}^{(\nu )}_i$ as a Michaelis-Metnten kinetics function^23^ of $\varvec{x}^{(\nu )}_j$, i.e., gene *i* is activated by gene *j*. The activation relation can be represented as a link in the gene regulatory network (GRN) with weight $w_{i,j}$. In our simulation we use GRNs with random links between the nodes, i.e., each pair of genes is connected with a constant probability in the form of an Erdős-Rényi network with an average degree of three. Finally, the GRN weights $w_{i,j}$ (for existing links) are randomly selected from the uniform distribution $\mathbb {U}(0,2)$. To create different cells from the same GRN, a random subset of genes in each cell are set as inoperative, i.e., their expression levels are set to zero. Specifically, in our simulations we randomly choose 5 out of the $N=200$ genes to be inoperative. The expression profile of each cell $\varvec{x}^{(\nu )}$ is generated by solving the GRN differential equations with the same initial conditions ($x_i^{(\nu )}(t=0)=0.5$) and evaluating the steady state using the ode45 MATLAB function.

Using this procedure, we create two distinct cohorts of cells, which differ by their GRNs. Each cohort *A* and *B* has its own weight matrix, $w^A_{i,j}$ and $w^B_{i,j}$. The cohorts has the same number of cells ($M=50$) and genes ($N=200$). The GRN of the two cohorts have a fraction $1-p$ of shared links, i.e., the GRN of cohort *A* has a *p* fraction of links that are not present in *B* and vice-versa. If $p=0$ the GRN of the cohorts are identical. As *p* is increased, the number of shared links is decreased, and when $p=1$ the GRNs of the two cohorts have no shared links.

After generating the cells in each cohorts, the sample-to-sample mean distances, *D*, between the cohorts is measured. It is defined as the mean distance between the cohorts divided by the sum of mean of distances inside each cohort, i.e.,

15 $$\begin{aligned} D = \frac{D_{AB}}{D_A + D_B}, \end{aligned}$$

where,

$$\begin{aligned}&D_A = \frac{2}{M(M-1)}\sum _{i,j\in A, i\ne j}\mathrm {Spearman}({\mathbf{}}{x}^i,{\mathbf{}}{x}^j)\\&D_B = \frac{2}{M(M-1)}\sum _{i,j\in B, i\ne j}\mathrm {Spearman}({\mathbf{}}{x}^i,{\mathbf{}}{x}^j)\\&D_{AB} = \frac{1}{M^2}\sum _{i\in A, j\in B}\mathrm {Spearman}({\mathbf{}}{x}^i,{\mathbf{}}{x}^j). \end{aligned}$$

When the cohorts are generated with the same GRN (i.e., $p=0$), $D=0.5$. As *p* is increased, *D* gets larger. The dependency of the GCL value on these parameters is presented in the Results section.

## Results

Here we describe the necessary pre-processing steps and the application of the GCL measure. Specifically, this includes: (1) Removal of abnormal cells, (2) Clustering analysis, and (3) Jackknife re-sampling.

### Removal of abnormal cells

In our previous work^15^, during the analysis of the single-cell transcriptomic data, we have noticed the significant sensitivity of the GCL towards single *abnormal* cells. The sensitivity is not only towards outliers, cells which are far away from the rest of the cells, but also for cells which are unusually similar to other cells, which we refer to here as ‘inliers’. The abnormality of the cells with respect to the majority of the cohort can be observed by examining the distribution of ‘distances’ (Spearman dissimilarity) between all the cells (Fig. 1a). While the cell-to-cell distances among most of the cells are distributed near a characteristic value, there are a small number of cells, associated with exceptionally large or small values, compared with the mean (red and blue shaded area respectively). Figure 1b shows the effects of outliers and inliers of a typical cohort of cells. The data is a cohort of short-term hematopoietic stem cells (ST-HSCs) from young C57 mice^19^. We measure the effect of each individual cell on the GCL, $\Delta \mathrm {GCL}_i$, defined as the relative difference between the GCL with and without cell *i* (see “Material and methods” section) For each cell we calculate the minimal distance between it and any of the other cells in the cohort. The minimal distance of an outlier would be relatively large, whilst the minimal distance of an inlier would be relatively low. When plotting $\Delta \mathrm {GCL}$ versus the minimal distance, a characteristic parabola-like shape is apparent (Fig. 1b), where both outliers and inliers have a significant relative effect on the value of the GCL (where outliers seem to be more significant then inliers). To ensure that the GCL is not biased due to the effect of a few outliers or inliers cells, we recommend filtering them out prior to the analysis. This can be done, for example, by looking into the distribution of distances between all the cells, and removing cells which have some distance which is two standard deviations above/below the mean distance between the cells (Fig. 1a). Note the inherent difference between outliers and inliers with respect to the cohort. When an inlier is present in the data, it means that there are two cells which are abnormally similar to each other. In this case, it is sufficient to remove only one of the cells in order to increase the homogeneity of the cohort. When an outlier is present, there is no arbitrary choice in the filtering process.

To examine this effect thoroughly, we introduce a simulated cell into a cohort of real cells and evaluate its $\Delta \mathrm {GCL}$, as follows. First, we chose two random cells from the cohort of real cells (blue and black circles in Fig. 1c). Then, we replace one of these cells with a simulated cell, constructed as a linear combination between the two cells, with a tuning parameter, $0\le \lambda \le 1$. When $\lambda =0$ the simulated cell is identical to the replaced cell. When $0<\lambda <1$, the simulated cell becomes more similar to the other cell. For each value of $\lambda$, we measure both the minimal distance and the $\Delta \mathrm {GCL}$ associated with the simulated cell. When the minimal distance decreases, the effect on the GCL increases (blue lines in Fig. 1d). We repeat this process for 10 random cells initiations.

We then perform a similar process on an outlier cell, by replacing one of the outlier cells (red circle in Fig. 1c) by a simulated cell, constructed as a linear combination between it and a random cell (the black circle in Fig. 1c). This time, in order to focus on the effect of the outlier with respect to the cohort and not on the similarity between two cells (the ‘inliers’ effect), we also remove the random cell from the analysis. This manipulation results in a simulated cell that, by tuning the $\lambda$ value, move on the trajectory between normal and outlier cell, i.e., when $\lambda =0$ the simulated cell is identical to the randomly selected cell, and when $\lambda =1$ the simulated cell is identical to the outlier cell. Figure 1d) shows that when the minimal distance increases, the effect of the simulated cell on the GCL significantly increases. Both the inliers and the outliers’ effects, which are shown in Fig. 1d) using simulated cells, demonstrate a similar pattern to the parabola shape observed for the real data.

Finally, we test the effects of outliers and inliers on three additional data-sets from independent sources^19,21,22^ (Fig. 1e). The figure shows the GCL values of cohorts of cells before and after the removal of outliers/inliers. While it is clear that their presence tends to increase the GCL, the most pronounced effect is visible in the ST-HSC cohort from Ref.^19^ (Fig. 1a). There, the presence of a few outlier/inliers (5) can cause the GCL of the jackknife group to dramatically increase to the maximum value (1). In fact, a bimodel distribution can be observed when the outliers/inliers are present, which represent jackknife groups with and without some of these unusual cells.

### The effect of clusters

Like other statistical inference tools, the GCL is also sensitive to heterogeneity in the data. A common source of heterogeneity is the presence of clusters in the cohort of cells. In this section, we detail how does the presence of clusters affects the GCL value, as well as provide recommended guidelines on how to filter out clusters. In general, clusters in the cohort tend to increase the GCL values, sometimes even to extreme values close to 1. This effect is analogous to the biases of Pearson correlation values calculated over heterogeneous data. The goal of the GCL is to provide a sense of the strength of the inter-dependencies between the genes. To this aim, we wish to calculate the GCL for isolated homogeneous cohorts, in order to avoid a type of Simpson’s paradox, where the presence of two or more clusters in the data affects the apparent relations between the genes (see “Discussion”).

**Figure 2:**
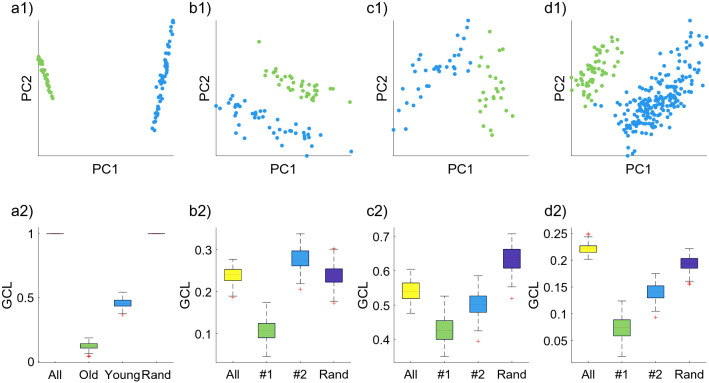
The effect of data heterogeneity on the GCL measure. (**a1**) PCA plot of a cohort of cells which contains artificial heterogeneity by combining MPP cells from two different mice from the same data-set (young and old mice from Ref.^20^). (**a2**) GCL values of the different groups of cells in (**a1**), including: the entire cohort (‘All’, $M=87$), only the cells from the old mouse (‘Old’, $M=34$), only the cells from the young mouse (‘Young’, $M=53$), and a subset of cells randomly selected from the entire cohort (‘Rand’, $M=34$). (**b1**) PCA plot of a cohort of old long-term hematopoietic stem cells (LT-HSCs) from^20^. The blue and green colors represent detected clusters of cells, which are labeled as groups #1 and #2, respectively. (**b2**) The corresponding GCL values of the groups in (**b1**). The total number of cells is $M=82$, the number of cells in group #1, #2 and the random subset is $M=37$, $M=45$ and $M=45$, respectively. (**c1**) Same as (**b1**) for MPP cells from^20^. (**c2**) The corresponding GCL values of the groups in (**c1**). The total number of cells is $M=59$, the number of cells in group #1, #2 and the random subset is $M=25$, $M=34$ and $M=34$, respectively. (**d1**) Same as (**b1**) but for LT-HSCs from old C57 mouse from^19^. (**d2**) The corresponding GCL values of the groups in (**d2**). The total number of cells is $M=256$, the number of cells in group #1, #2 and the random subset is $M=58$, $M=198$ and $M=198$, respectively. Box plots represent the distributions of GCL values calculated for 100 jackknife realizations, each consists of $80\%$ of the cells and calculated using $m=50$ random choices of gene group divisions. The data consists of the top 500 expressed genes. These results demonstrate how heterogeneity in the data can cause spurious GCL values, while dividing the data into its natural clusters yield distinct GCL values. Random groups are comprised of random cells from the entire cohort, with the number of cells equals to the size of group #2. Centre line, median; box limits, upper and lower quartiles; whiskers, 1.5 interquartile range; points, outliers.

Figure 2a1–a2 shows this effect in a heterogeneous cohort, artificially induced by combining multipotent progenitors (MPP) cells from two different mice (ages 3 months (‘young’) and 22 months (‘old’)) (from Ref.^20^, see “Material and methods”). Calculating the GCL over the entire cohort yields an extreme value which is very close to 1, whereas the GCL values of each separate group (young and old) have much more sensible values, lower than 0.5 (Fig. 2a2). To verify that this change of the GCL values is not due to the change of sample size (number of cells), we calculate it also over a fourth group, which is comprised of a random assortment of the same number of cells as one of the clusters, selected from both the young and old groups. Figure 2a2 shows that this group also has an artificially large GCL value. Figure 2b–d also show the effect of heterogeneity, but for naturally occurring clusters (from Ref.^19,20^). The clusters are marked with blue and green colors. The effect of the clusters on the GCL is evident (although less dramatic than the artificial case in a). A mixed cohort will tend to have larger GCL values than any of the separate clusters.

**Figure 3:**
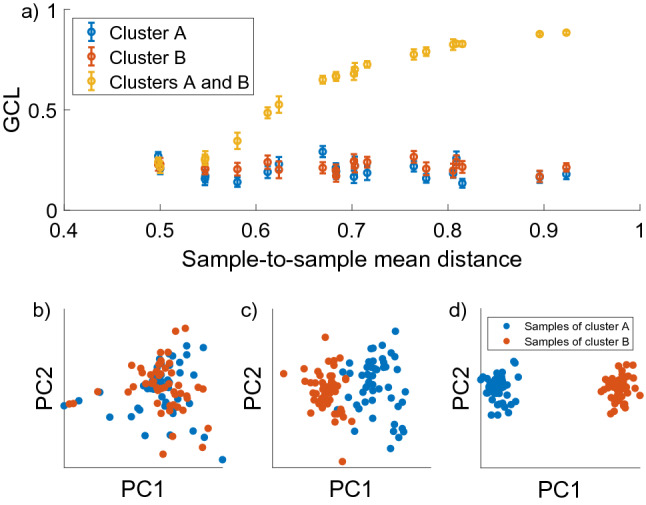
The effect of data heterogeneity on the GCL measure in simulated gene expression data. (**a**) The GCL as a function of the sample-to-sample mean distances between two simulated cohorts *A* and *B*. The GCL is calculated separately for cohort *A*, cohort *B* and the joint cohort *A* and *B* (blue red and yellow colors respectively). The dots represent the mean value of the GCL and the error bars represent the standard deviation of the 20 jackknife realizations. The differences between the GRN are controlled by the parameter $0<p<0.2$ (see “Material and methods” ). As *p* increases, the sample-to-sample mean distances,*D*, increases as well. While the value of the GCL of each individual cohort remains stable, the value of the combined cohort increases to 1 due to the artificial heterogeneity in the data. (**b**–**d**) PCA visualizations of the two cohorts for three different values of *D* ($p = 0,0.04,0.2$ with $D=0.50063, 0.54042, 0.8583$ respectively). The increased separation is visibly apparent. Note that for $p=0$, $D\approx 0.5$ and the clusters of the cohorts are not distinct.

We also verify this effect using numerical models of synthetic gene expression data (see “Material and methods” ). Two cohorts, *A* and *B*, of increasingly differentiated gene regulatory network are generated, and the GCL of them is measured (Fig. 3). The cohorts have the same number of cells ($M=50$) and genes ($N=200$). When the GRN of the two cohorts is identical, the GCL value of each cohort approximately equals to the GCL of the joint cohorts, which is comprised of all the cells from both *A* and *B*. As the differences between the GRN of the cohorts increase, the GCL of each individual cohort remains stable. This shows that the GCL, which measures the general dependencies between the genes is not sensitive to small-scale differences. However, the GCL of the combined cohort increases. This is due to the heterogeneity in the data, which is a confounding factor that artificially increases the GCL, similar to what is observed in real data.

We have shown here how the presence of clusters creates artificial inflation of the GCL value. In “Material and methods” , we detail our proposed recommended method of how to detect and filter clusters in the data.

### Jackknife re-sampling procedure

Generally, the stability of the GCL calculation, i.e., how sensitive is it to the exact cell composition of the cohort, is related to two factors: the homogeneity of the cell population, and the sample size (number of cells in the analyzed cohort). To evaluate the stability of the GCL for a given cohort of cells, we apply the jackknife re-sampling procedure^26^. Specifically, we randomly re-sample *s* sub-sets of *k* cells, with repetition, and calculate the GCL of each of them. If the cell population is substantially homogeneous, the variance of the GCL values calculated over the different sub-sets will mainly reflect the finite size effect. In contrast, if the cell population includes a small number of outlier cells (see “Removal of abnormal cells”), they will be sampled and affect the GCL values in only some of the sub-sets, increasing the variability of the GCL values. Therefore, the jackknife procedure is well-suited for the stability estimation of the GCL.

However, re-sampling sub-sets of cells, with repetitions, from a finite cohort introduces the issue of the choice of the overlap level between the different sub-set. Thus, the predetermined value of the *k* parameter evidently affects the variance of the GCL values. For large values of *k*, the overlap between the different sub-sets is high, and subsequently the variance of the GCL values will be relatively small. On the other hand, for small values of *k* (small number of cells in each sub-set), the GCL results are highly vulnerable to finite-size noise and hence the variance of the GCL values will be relatively large.

**Figure 4:**
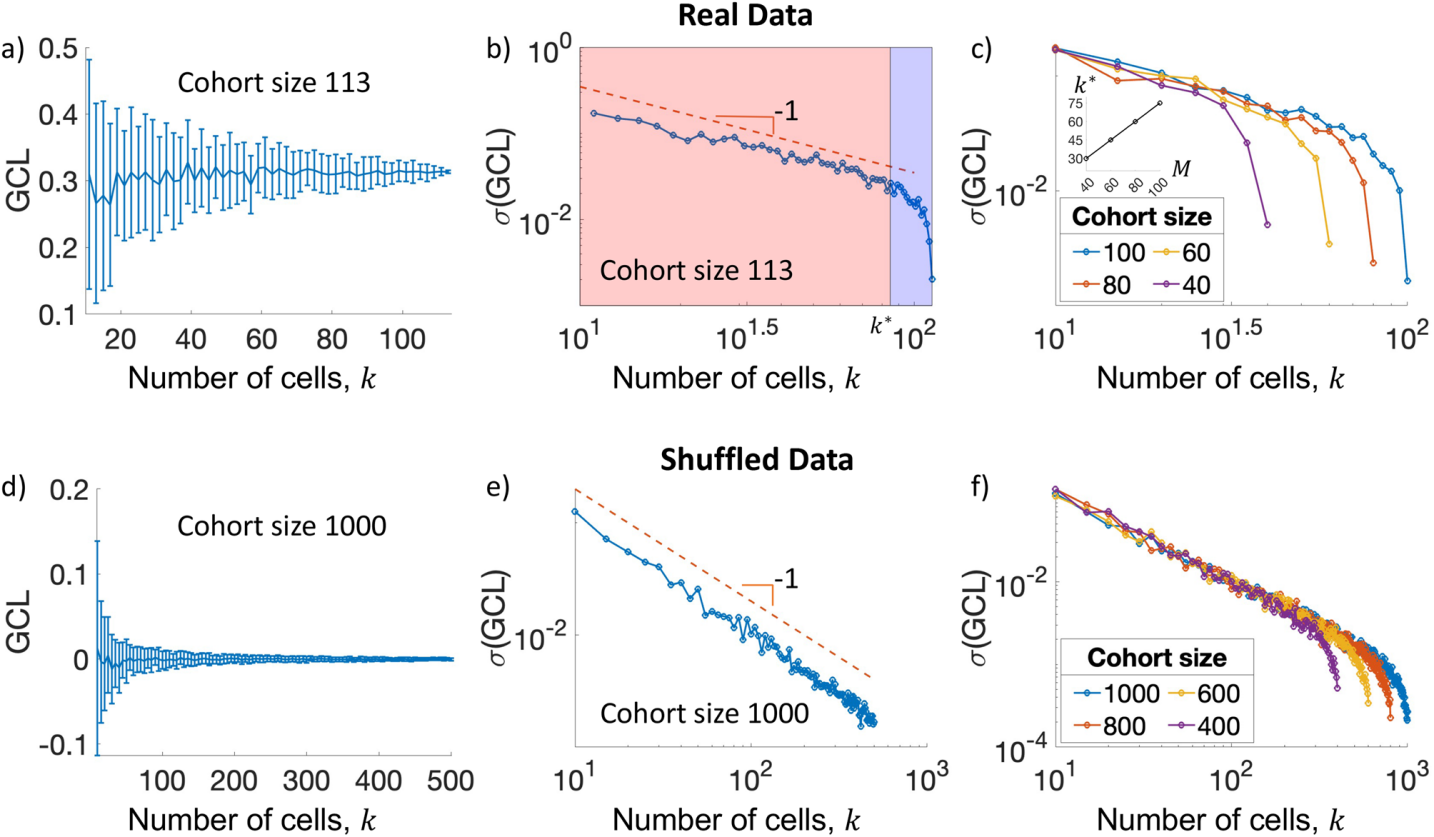
The effect of jackknife sample size on the GCL variability. Given a cohort of $M=113$ cells (as detailed in the text), we re-sample at random $s=40$ subsets with $k<M$ cells and calculate the GCL of each subset. (**a**) Mean and standard deviation, $\sigma (GCL)$, of the GCL distributions as a function of *k*, represented by the center and the length of the error bars, respectively. (**b**) The standard deviation, $\sigma (GCL)$, as a function of *k*. The red area, where $k<k^*$ is dominated by the finite-size effects. When $k>k^*$, the $\sigma (GCL)$ drops much more rapidly, due to the large overlap between the jackknife subsets. (**c**) The same analysis as in (**b**) but for four cohorts of sample-size $M = 40,60,80, 100$, where each cohort is chosen at random from the total 113 real data cells. The inset shows $k^*$ versus the cohort size, *M*. The linear relationship suggests that an optimal *k* value is approximately $75\%$ of the sample-size. (**d**–**f**) Similar analyses as in (**a**–**c**) but for shuffled data with a cohort size of $M=1000$. In this case, the average GCL is 0. The optimal value of $k\approx 75\%\times M$ still holds. We use $m=20$ for all GCL calculations.

To test this effect, we systematically calculate the GCL values with the jackknife procedure using different values of *k*. The data presented in Fig. 4 contains the gene expression profiles of $N=2000$ genes (with the largest mean expression) from $M=113$ LT-HSCs from a young C57 mouse^19^.

Figure 4a shows the GCL distributions of the jackknife realizations for different values of *k*. While the average GCL is reasonably constant of *k*, the distributions are increasingly narrower for larger values of *k* (specifically, when $k=113$, all the subsets are identical and their GCL values converge to a single value). When plotting the standard deviation of the GCL distributions, $\sigma (\mathrm {GCL})$, versus the number of cells, *k*, on log-log plot, two regions can be observed (Fig. 4b). For $k<k^*$, where $k^*\approx 85$, $\sigma (\mathrm {GCL})\sim k^{-1}$ (linear relation on the log-log plot at the red colored zone). For $k>k^*$, $\sigma (\mathrm {GCL})$ rapidly decreases as *k* approaches the cohort size (blue colored zone). The two regions correspond to the two effects mentioned above. For small values of *k*, the decrease of $\sigma (\mathrm {GCL})$ is mainly due to the increased sample size of each jackknife realization. For large values of *k*, the large overlap between the re-sampled subsets becomes the dominant effect.

These results suggest that $k=k^*$ may be considered as an optimal choice, as it decreases the ‘finite size effect’ with a minimal ‘overlap effect’. Next, we test how the value of $k^*$ depends on the cohort size. We repeat the same analysis as in Fig. 4b for smaller cohort sizes (selected from the same data-set). Figure 4c shows that the cross-over value, $k^*$, between the linear decay (on the log-log scale) and the rapid decrease of $\sigma (\mathrm {GCL})$ can be approximated as $\approx 75\%$ of the cohort size.

We repeat the analysis for shuffled data, with a much larger sample size ($M=1000$). The shuffling procedure removes the relationship between the genes, causing the average GCL to be equal to 0 (Fig. 4d). By having a cohort size which is much larger than *k*, the overlap effect is negligible and the sample-size effect is dominant. As shown in Fig. 4e, the sample-size effect yields $k^{-1}$ dependency of the standard deviation of the GCL, similar to the observation in Fig. 4b. When analyzing cohorts of shuffled data with different sizes, we find that the $k^*$ value is about $\approx 75\%$ of the cohort size (Fig. 4f).

To conclude, when analyzing the stability of the GCL results in other cohorts, we recommend to repeat the analysis presented here to find the optimal value of *k* for the jackknife procedure. Otherwise, we would recommend to choose $k\approx 75\%\times M$ as a default value.

### GCL script description

We publish on GitHub^27^ an open-source code package written in Python 3.9 which includes a tutorial and working example of the GCL calculation as well as a GCL library. The tutorial demonstrates the calculation of the GCL for two scRNA-seq data sets of LT-HSCs from young (3 months) and old (22 months) mice from Ref.^19^. These data sets are in a .CSV format, where rows represent the expression levels of the 3000 genes with the highest mean expression, and columns represent individual cells. The output of the tutorial is a figure showing the distributions of the jackknife realizations values of both scRNA-seq data sets. The figure compares the two histograms showing how different parameters of the input (‘percentage’, ‘jackknife realizations’ and ‘divisions number’) influence the results.

**Figure 5:**
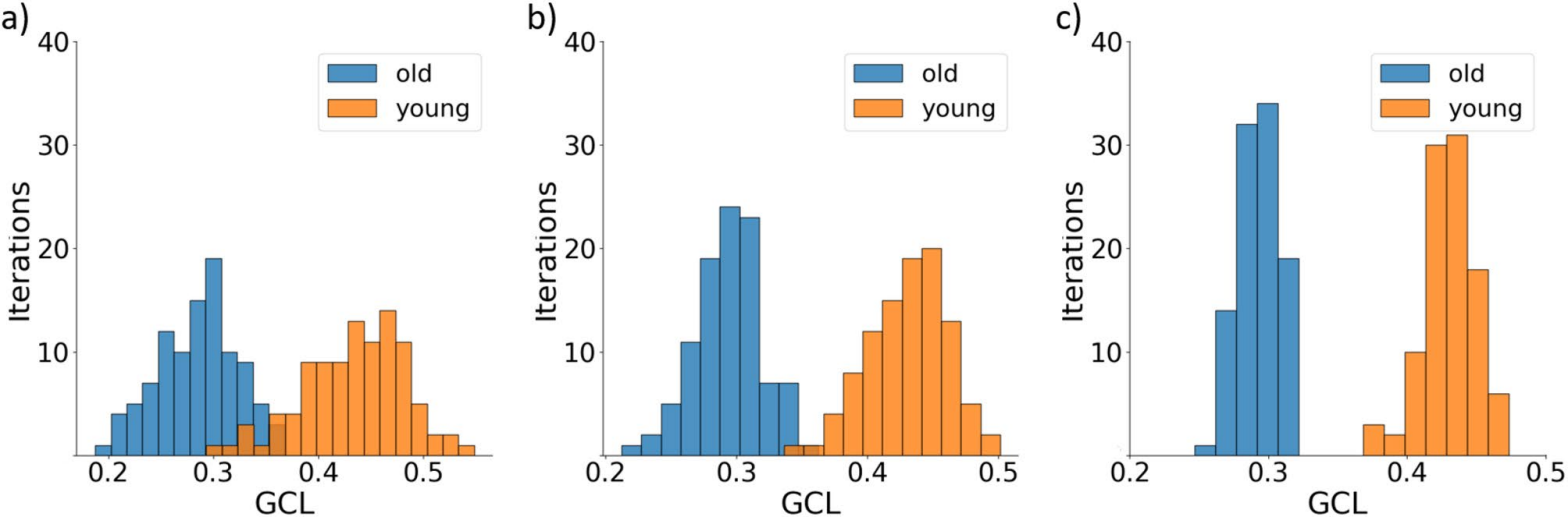
Demonstration of the GCL implementation. An example of the use of the GCL Python script. Two cohorts of gene expression tables of LT-HSCs^19^ from young and old organisms are compared with respect to their GCL values. The user chooses the number of jackknife realizations, *s* (in this case $s=100$), and the percentage of re-sampled cells in each realization from the entire cohort. (**a**) $50\%$, (**b**) $70\%$, (**c**) $90\%$. The function plots the values as a histogram. The characteristic decrease in GCL values with age is apparent^15^. Increasing the percentage of cells decreases the variance of the GCL.

The library can be imported into an existing Python project or executed as a stand-alone program to calculate the GCL for given data (vector or GCL values for the jackknife realizations). Different features of the GCL calculations can be manually chosen. For example, Jackknife_percentage determines the fraction of cells in each jackknife realization from the entire cohort, *k*/*M*; and Jackknifes determines the number of jackknife realizations, *s*. Figure 5 shows a typical output of the Python script for a cohort of LT-HSC cells. The GCL distributions become narrower for larger values of *k*, where *k*/*M* is set to be 0.5, 0.7 and 0.9 in Fig. 5a–c), respectively. A second script, which only calculates the GCL using MATLAB is also freely available^28^.

## Discussion

In this manuscript, we present general guidelines and recommended practices on how to appropriately utilize the GCL method on scRNA-seq datasets. A main consideration when applying any bulk analysis method to a cohort of cells, such as co-expression analysis or the GCL, is to ensure that the analyzed cohort is as homogeneous as possible. This does not mean that the transcription profiles of all the cells must be identical, or very similar to each other, but rather that the cell-to-cell variability should not be dominated by a small number of outlier cells or by the presence of distinct clusters. The presence of outliers or clusters may lead to spurious results when applying measures of gene–gene interrelations, such as co-expression analysis or GCL. Therefore, we consider the removal of outliers and clusters as a standard pre-processing procedure and a necessary step in analyses that focus on the interrelations between genes.

‘Inliers’, i.e., cells which have unusually similar transcription profiles, is another condition that should be taken into account when calculating the GCL. Two very similar cells could be present due to biological effects, for example, if the sampling corresponded with a recent division of the cell. We show that the GCL may be disproportionately affected by such cases in the cohort, with a tendency to artificially increase its value. We therefore recommend to view such cells as another form of heterogeneity, and to remove one of them before the calculation of the GCL.

We recommend to accompany the calculation of the GCL with a jackknife procedure, where the GCL is calculated for a number of re-sampled sub-sets of cells, yielding a distribution of values. There are two benefits to this step. First, it can serve as an additional layer for testing the homogeneity of the analyzed cohort. Specifically, if the bulk calculation of the GCL is disproportionately affected by individual cells, the subsets that include it will have elevated GCL values that may be observed by examining the distribution (see e.g. Fig. 1e). Such cells may be missed in the initial pre-processing steps described above. Note, however, that the jackknife procedure is not suited for detecting the presence of clusters, since this kind of heterogeneity is preserved in the re-sampled subsets, as demonstrated in Fig. 2. Second, when comparing GCL values from two different cohorts, the jackknife distributions are essential. For this purpose, a rational choice of the re-sampling size (the value of *k*) is required. We propose a methodology to balance between two extremes: a too small value of *k* introduces statistical noise due to small sample-size, whereas a value of *k* that is too close to the cohort size will not generate the representative variability of the GCL, due to the high overlap between the re-sampled sub-sets. Additionally, the jackknife re-sampling is an effective way to eliminate the potential bias due to a different number of cells when comparing two or more cohorts of different sizes, by calculating the GCL over the same size of re-sampled subsets.

The recommendation to apply the GCL on homogeneous cohorts may seem counter-productive to the usefulness of scRNA-seq to reveal the heterogeneity of the data. Indeed, the heterogeneity can discover hidden information, such as identifying new sub-types of cells. Importantly, we distinguish between two types of heterogeneity: (1) heterogeneous cohorts that contain different cell types/sub-types, or individual outliers/inliers cells; (2) the natural cell-to-cell variability of gene expression profiles among cells of the same tissue, cell type and biological conditions. The goal of the GCL is to unveil underlying order, in terms of gene–gene coordination, from the latter type, i.e., natural cell-to-cell variability. For the GCL analysis, like other correlation analysis methods, heterogeneity of the first type in the analyzed data may be detrimental. It has the proclivity of generating spurious relations, e.g., strong observed correlations due to confounding factors (Simpson’s paradox). Therefore, the pre-processing steps should minimize the first type of heterogeneity while preserving the natural cell-to-cell variability.

The added value of the GCL with respect to the traditional bottom-up approaches can be illustrated by the following example. Consider an experiment to test the effect of a given condition by comparing the scRNA-seq data of control and case cohorts. We can divide the apparent differences between these two cohorts into two kinds. Gene-specific differences are ‘small-scale’. They can be detected as deferentially expressed genes (DEGs) between the case and control, or as ‘differential gene–gene interactions’, which may indicate an alteration of specific elements in the underlying regulatory mechanism. The second type is ‘large-scale’ differences, i.e., not gene-specific. For example, during aging, cells accumulate stochastic genetic and epigenetic damage that affect each individual cell differently. The group-effect of such random damage is not observed in the same genes across different cells, but rather may be observed as a general decline of the gene-to-gene dependency level. The GCL is a measure of this kind.

In practice, when comparing two cohorts we recommend first to detect any small-scale differences between them using the traditional approaches, e.g. filtering DEGs between them. Then, the GCL can be applied on the remaining genes, i.e., the non-differentially expressed genes. This is beneficial in two ways. On the one hand, it rules out the potential bias due to the effect of the DEGs on the GCL. On the other hand, if there are observed significant differences between the GCL values calculated over the non-differentially expressed genes, then they may be interpreted as a result of large-scale alterations.

As a final remark, the proposed filtering process presented here uses auxiliary algorithms (Silhouette, Spearman-based outlier removals, etc) which might not be best suited in all cases. There is a plethora of cluster identification and outlier removal algorithms in the scientific literature, and it is certainly possible that the use of one of them would be more advantageous than the ones presented here for other sc-RNA datasets. For example, UMAP can replace *t*-SNE in the visual inspection step, DBSCAN can be used for clusters identification, and the Spearman matrix can be replaced by other dissimilarity measures, such as Euclidean distance, normalized mutual information, adjusted Rand index, Fowlkes–Mallows index or the Jaccard index. The goal of this manuscript is to demonstrate the sensitivity of the GCL to the structure of the data and propose possible solutions.

The GCL is therefore a complementary measure to the existing classical tool-set of gene-expression analysis. We hope that the GCL method, along with the recommended guidelines in this manuscript, be useful in the future analysis of scRNA sequencing.
